# Case Report: Personalized Therapeutical Approaches with Lenalidomide in Del(5q): A Case Series

**DOI:** 10.3389/fonc.2022.866470

**Published:** 2022-03-31

**Authors:** Anna Stein, Anne Sophie Kubasch, Claudia Haferlach, Uwe Platzbecker

**Affiliations:** ^1^ Department of Hematology, Cellular Therapy and Hemostaseology, Leipzig University Hospital, Leipzig, Germany; ^2^ Munich Leukemia Laboratory, Munich, Germany

**Keywords:** MDS del(5q), lenalidomide, case-report, del(5q), t(2;5)

## Abstract

Myelodysplastic Syndrome (MDS) with del(5q) represents a unique WHO entity, which is often treated with lenalidomide according to standard clinical practice. Guidelines concerning treatment duration have thus far not been implemented, but rather comprise an indefinite therapy until loss of response. This review presents three red blood cell (RBC) transfusion-dependent MDS with del(5q) cases, starting with one rare case with an unbalanced translocation t(2;5), involving the breakpoint of del(5q) and loss of the 5q15-5q31 region. To the best of our knowledge, no comparable case has been described before with a response to lenalidomide. Strikingly, treatment-induced and maintained cytogenetic complete remission (cCR) in this patient. Furthermore, we report two cases of classical del(5q), in which lenalidomide was interrupted after a short period of lenalidomide therapy at the time cCR was achieved. Despite drug holiday cCR was maintained for seven and nine years, respectively. Then del(5q) re-emerged in the absence of novel molecular aberrations and re-treatment with lenalidomide could again achieve cCR in both cases. Together, this series presents three cases of personalized therapy of MDS with del(5q).

## Introduction

MDS namely describes a disease, characterized by dysplastic bone marrow cytomorphology leading to peripheral blood cytopenia and possible transformation to acute myeloid leukemia (AML). The typical hallmark of this disease is its huge diversity and heterogeneity, not only in terms of cytogenetical and cytomorphological changes, but also in its individual clinical courses and risk of disease progression. The current WHO classification distinguishes thirteen distinct entities of MDS, including MDS with del(5q) to the loss of 5q31 or 5q32 as the common region of deletion (CDR) (1). as a subgroup of MDS. According to the International Prognostic Scoring System (IPSS) cytogenetic risk categories MDS with del(5q) is classified as lower risk in most cases. Erythroid insufficiency and favorable transformation risk in the absence of a *TP53* mutation represent the main clinical characteristics. Patients mostly suffer from reduced quality of life due to anemia and dependency on RBC-transfusions. Lenalidomide represents the first-line treatment for transfusion-dependent MDS with del(5q). Evidently, it leads to transfusion independence in two-thirds of patients while cCR is seen in 50% of cases (2–4). Patients treated with lenalidomide display no increased risk of disease progression to AML (2, 5). As of now, medical guidelines offer no consensus regarding the duration and possible drug holiday of treatment with lenalidomide, which can be associated with hematological and economic toxicities (6).

The pharmacological effect of lenalidomide acts *via* a dual mechanism: First by selectively inhibiting the growth of del(5q) positive progenitors through ubiquitination by the E3 ubiquitin ligase cereblon (7). Second restoration of erythropoiesis is achieved by inactivating p53 in erythroid progenitor cells and stabilizing EPO receptors (2, 8). Nevertheless, progression or loss of response occurs in 50% of all cases within two years of treatment with lenalidomide (4). Before and during lenalidomide treatment regular controls of *TP53* alterations are obligate to detect clonal evolution well enough in advance. Currently, it is recommended to treat patients continuously until loss of response or disease progression. We here present three cases of patients including two where lenalidomide was stopped as a personalized concept to avoid toxicity or potentially promote clonal evolution.

## Case Description

**Figure 1:**
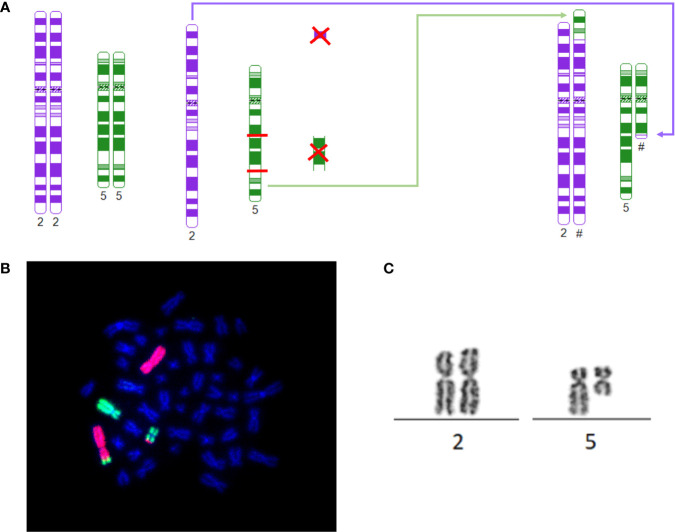
**(A)** Schematic depiction of breaking points, rearrangement and derivates of 46, XX, der(2)t(2;5)(p23;q31), der(5)t(2;5)(p25;q15)[21]: The model was constructed using http://www.cydas.org/OnlineAnalysis/ (9). **(B)** 46, XX, der(2)t(2;5)(p23;q31), der(5)t(2;5)(p25;q15)[21]: Whole chromosome painting of case 1 with karyotype: 46, XX, der(2)t(2;5)(p23;q31), der(5)t(2;5)(p25;q15). Chromosome 2 in red, chromosome 5 in green. **(C)** t(2;5): Part of the karyogram of case 1 depicting t(2;5) as part of 46, XX, der(2)t(2;5)(p23;q31),der(5)t(2;5)(p25;q15)[21].

Case 1 presented with a rare del(5q) as a result of an unbalanced translocation, leading to a partial loss of 5q (**Figure 1**). The patient was 47 years of age at the time of diagnosis, presenting with progressive macrocytic anemia (Hb *4,1* mmol/l). The patients karyotype displayed 46,XX, der(2)t(2;5)(p23;q31),der(5)t(2;5)(p25;q15) in 21 of 31 analyzed metaphases with an additional 47, XX,+8 in three metaphases. Having a normal count of blasts in the bone marrow and no peripheral blasts, she was scored within the intermediate-1 risk group according to IPSS. Supposing the same underlying cellular path mechanism as in regular del(5q) MDS, a therapy with lenalidomide (10 mg daily, day 1 to 21) was initiated. Although in this case no classic MDS with del(5q) was evident, cCR and transfusion-independency could rapidly be achieved after 6 months of treatment. However, novel mutations within *ASXL1* (VAF 7%) and *DNMT3A* (VAF 10%) emerged, while the pathological karyotype remains at detection level (1/20 metaphases). Since then, therapy with lenalidomide is been continued with ongoing complete hematological remission.

**Figure 2:**
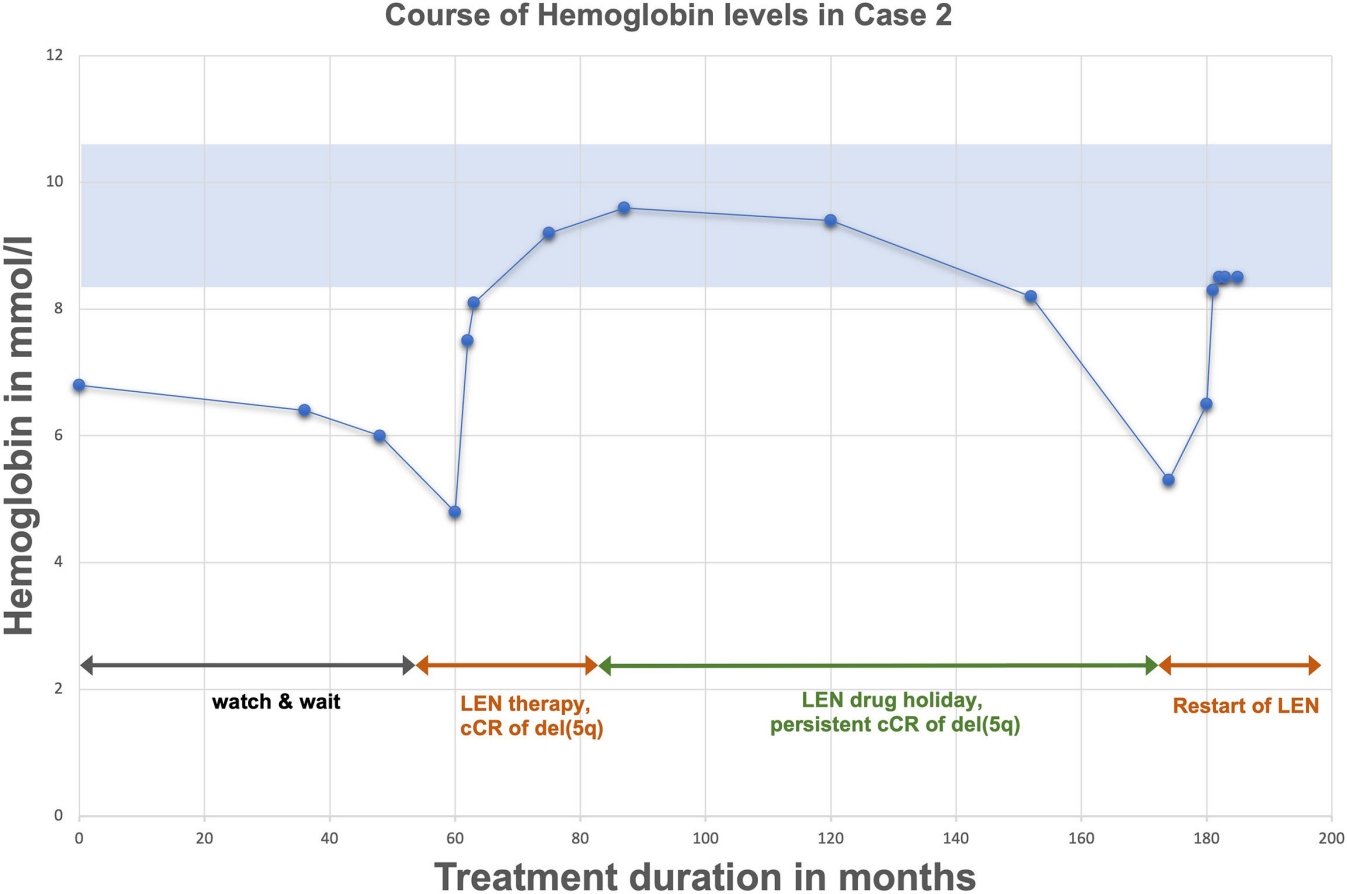
Exemplary course of Hemoglobin (case 2) upon treatment and drug holiday with lenalidomide.

Case 2 represents a 58-year-old woman first diagnosed with MDS del(5q) in 2006 due to anemia. At this time average hemoglobin-levels (Hb) were measured at *6,8* mmol/l (**Figure 2**). Cytogenetical analysis revealed an isolated del(5q) with no further aberration, molecular genetics showed no mutations, especially no *TP53* mutation. Prognosis was scored as intermediate-1 according to IPSS. At first, the patient was kept under constant surveillance (watch & wait) without need for treatment due to little risk of malignant transformation and no need for RBC transfusions. In 2011 anemia progressed (Hb 4,8 mmol/l) with new RBC transfusion dependency, despite cytogenetic and molecular characteristics remaining unchanged (isolated del(5q)). In line with the current guidelines a therapy with lenalidomide with a daily dose of 10 mg for day 1-14, following a drug holiday for seven days was started. Two months later hemoglobin levels normalized. After three months the patient showed cCR in bone marrow analysis. Lenalidomide was continued for two years with cCR being maintained. During this period no further genetic changes accumulated as assessed by regular cytogenetic and molecular analysis incl. the absence of a *TP53* mutation. After two years of treatment and persistent cCR, treatment was terminated and switched to active surveillance with the patient remaining in cCR for close to seven years. In 2020 (six years after stopping) anemia resurfaced (Hb *5,1* mmol/l) alongside a cytogenetic relapse del(5q) in 5 out of 20 metaphasis. Due to renewed disease activity another period of lenalidomide therapy was successfully initiated.

The final case displays a quite similar course of a 57-year-old woman. The patient was first diagnosed in 2010 with MDS del(5q) and likewise scored as intermediate-1 risk, according to IPSS. At the time of diagnosis hemoglobin-levels were already lower than in case 2 (Hb *4,7* mmol/l), so that RBC transfusions became necessary shortly after diagnosis. The treatment with lenalidomide was initiated immediately, according to the dosage regimen mentioned in case 2. CCR was reached after five months of treatment and maintained. Again, lenalidomide was paused, after one year of stable cCR, with cCR being maintained for nearly nine years without further treatment. During this time regular bone marrow analysis showed no further alterations. In 2021 hemoglobin-levels began to drop down to *4,4* mmol/l and the bone marrow puncture revealed the re-emergence of del(5q) in 7 out of 35 metaphasis. Following, another cycle of lenalidomide therapy at a dosage of 5 mg was initiated, leading to quick reconstitution of hemoglobin levels.

## Methods

As previously described according to standard methods, chromosome banding analysis and FISH (10), as well as next-generation sequencing (11) were performed by MLL – Munich Leukemia Laboratory.

## Discussion

The most frequently observed genetic changes within MDS is a del(5q). Haploinsufficiency causing casein kinase CK1α dependency of erythroid cells is proposed as the most likely pathomechanism (12). The regular del(5q) has been narrowed to a CDR including 5q32 and 5q31 (13). The first patient presented with an unusual complex rearrangement with breaks occurring at 2p23 and 2p25 as well as in 5q15 and 5q31 with fusion of 2p23 and 5q31 and 5q15 and 2p25, leading to loss of the segments 2p23 to 2p25 and the segment 5q15 to 5q31, thus preserving parts of 5q31 and 5q32. *Cereblon* (CRBN) poses the target of immunomodulatory drugs, such as lenalidomide and acts as substrate receptor of the CRL4^CRBN^ E3 ubiquitin ligase, therefore mandating degradation. *Cereblon* is located on 3p26.2 and therefore spared from the complex karyotype displayed in case 1 (14). Although the CDR of MDS del(5q) was unaffected the patient still presented with the typical clinical features of del(5q) phenotype, such as macrocytic anemia, erythroid hypoplasia, normal or elevated platelet count and hypolobulated megakaryocytes (15). In terms of RBC, she responded well to lenalidomide, which is common for 26,9% of non-del(5q) as well (16). However, achieving CR in non-del(5q) upon lenalidomide treatment is very rarely observed (2, 16). Contrary, controls within our patient showed a rapid achievement of cCR upon lenalidomide treatment. At the time no comparable case of this particular karyotype responding to lenalidomide has been published. Following profound research of literature, the only relatable data describes an analysis of a cohort inheriting multiple interstitial deletion, one of which was 5q15-5q31 (17). However, most of these deletions included loss of 5q31 as the CDR of MDS del(5q) as well, which leaves our particular case unique so far. Although MDS del(5q) is usually associated with favorable risk of progression, data exist of AML-patients, displaying a karyotype including loss of 5q31 (15, 18, 19). Referring to the herein described unbalanced translocation, it is of note, that the specific CDR 5q31 of MDS with del(5q) remains partially preserved. However, 5q31represents the distinct breaking point. Thus, a fusion kinetic could be conceivable, as well.

Since 2005 lenalidomide has been FDA approved for patients with lower risk MDS del(5q) and failure of treatment with Erythropoiesis stimulating agents or dependency to RBC-transfusion (20). Lenalidomide is a very efficient treatment with high rates of CR and cCR with independence to RBC-transfusion (5). At the same time, lenalidomide does not seem to increase the rates of progression to AML (6). Most adverse effects were reported during first weeks of treatment most frequently in form of severe cytopenia (like thrombocytopenia and neutropenia) and could be resolved by dose reduction or disruption (3, 21). The greater challenge in clinical use of lenalidomide is posed by the fact, that a majority of the treated patients develop drug resistance upon time, due to PP2Acα-overexpression or evolving *TP53*-mutation (2). Therefore, regular controls of *TP53* alterations are obligate. The median duration of response for isolated del(5q) is about 2.3 years (4). All three cases showed no alteration of *TP53* at time of first diagnosis, nor during therapy with lenalidomide. In both cases treatment was paused after several months of stable cCR. Interestingly despite pausing lenalidomide, cCR could be maintained for more than 7 and 9 years without dependency to RBC-transfusions. Cases of prolonged transfusion independence and absence of cytopenia upon cessation of lenalidomide treatment have been described before (22–26); the longest being 36 months (26). Giagounidids et al. report a case in which a treatment period of only 28 days was sufficient to achieve transfusion independence for 21 months (23). However, in most of these cases treatment success was referred to as the absence of transfusion dependency or severe cytopenia, but in most of the cases no stringent cytogenetic remission could be obtained. Notably, none of the cases describes the re-initiation of treatment leading to a second cCR, like shown in the herein described cases. These similar cases emphasize the need of addressing the question of how long lenalidomide treatment should last. Furthermore, it poses the concern, if a strict cCR will be obligate for pausing therapy. We therefore suggest that this strategy should be explored in a prospective clinical trial.

To our knowledge this is the first reported case of an unbalanced translocation between chromosome 2 and 5, leading to loss of 5q15-5q31 and responding to lenalidomide.

## Conclusion

Our cases suggest that a drug holiday (like in CML) of lenalidomide upon reaching stable CR might be advisable to avoid drug resistance or clonal evolution. A clinical trial recommending the duration of lenalidomide therapy among MDS del(5q) would be of high interest.

## Ethics Statement

Ethical review and approval were not required for the study on human participants in accordance with the local legislation and institutional requirements. The patients/participants provided their written informed consent to participate in this study.

## Author Contributions

AS and AK collected data for the study, searched the literature, and wrote the manuscript. CH provided part of the data, illustrations, and wrote methods. UP conceived the manuscript, reviewed topic presentation, structure of the manuscript, illustrations and photographs. All authors contributed to the article and approved the submitted version.

## Conflict of Interest

The authors declare that the research was conducted in the absence of any commercial or financial relationships that could be construed as a potential conflict of interest.

## Publisher’s Note

All claims expressed in this article are solely those of the authors and do not necessarily represent those of their affiliated organizations, or those of the publisher, the editors and the reviewers. Any product that may be evaluated in this article, or claim that may be made by its manufacturer, is not guaranteed or endorsed by the publisher.

## Abbreviations

MDS, Myelodysplastic syndrome; cCR, cytogenetic complete Remission; RBC, red blood cell; AML, acute myeloid leukemia; IPSS, International Prognostic Scoring System; CDR, common region of deletion; Hb, hemoglobin.
